# MicroRNA320e Augments the Synthetic Lethality of Olaparib by Regulating the PI3K-AKT-mTOR Pathway

**DOI:** 10.32604/or.2026.081656

**Published:** 2026-08-13

**Authors:** Wei Zheng, Qianlong Meng, Yunhan Deng, Ruizhen Liu, Siyu Bai, Longyu Jia, Jing Wang, Huimin Bai

**Affiliations:** 1 Department of Gynecology, Fuxing Hospital, Capital Medical University, Beijing, China; 2 Department of Diagnostics of Clinical Laboratory, Zhejiang Hospital, Hangzhou, China; 3 Beijing Key Laboratory of Mental Disorders, National Clinical Research Center for Mental Disorders & National Center for Mental Disorders, Beijing Anding Hospital, Capital Medical University, Beijing, China; 4 Advanced Innovation Center for Human Brain Protection, Capital Medical University, Beijing, China

**Keywords:** MicroRNA320e (miR-320e), poly ADP-ribose polymerase inhibitors (PARPi), PI3K-AKT-mTOR, olaparib, fibronectin 1 (FN1)

## Abstract

Objectives: Given the increasing drug resistance in ovarian cancer (OC), the use of poly ADP-ribose polymerase inhibitors (PARPi) for treating homologous recombination repair defects (HRD) has encountered new challenges. MicroRNA320e (miR-320e) exerts a negative regulatory role in the progression of multiple cancers. This study aimed to investigate the association between miR-320e and drug resistance in ovarian cancer. Methods: The Cell Counting Kit-8 (CCK-8) assay, migration and invasion assays, and colony formation assay were employed to evaluate the proliferation, migration, and invasion abilities of cells. Western blot (WB) analysis was used to verify the expression levels of proteins related to the relevant signaling pathways in cells. The xenograft subcutaneous tumor model was established to investigate the effect of miR-320e on *in vivo* tumor growth. Immunohistochemistry (IHC) and fluorescence *in situ* hybridization (FISH) assays were performed to detect the expression levels of related proteins and miR-320e in tumor tissues from patients and animals, respectively. Results: miR-320e was overexpressed in both A2780 and SKOV3 cells. The results showed that transfection with miR-320e significantly reduced cell proliferation, invasion, and migration, while enhancing autophagy and apoptosis. Additionally, the PI3K-AKT-mTOR signaling pathway was significantly inhibited in the treatment groups. In nude mouse models, overexpression of miR-320e also significantly suppressed tumor growth. These findings indicate that overexpression of miR-320e enhances the sensitivity of OC cells to olaparib therapy. Conclusion: In conclusion, miR-320e overexpression significantly inhibits the malignancy of ovarian cancer and increases the sensitivity of ovarian cancer cells to olaparib.

## 1 Introduction

Phosphatidylinositol 3-kinase (PI3K) is a family of lipid and serine/threonine kinases with a heterodimeric structure, consisting of a p110 catalytic subunit and a p85 regulatory subunit [1]. As a downstream effector in the PI3K pathway, it can be activated by hormones, cytokines, and growth factors. Activated PI3K generates PIP2 and PIP3, which in turn phosphorylate AKT, one of its downstream signaling molecules [2]. Activated AKT regulates the kinase activity of the mammalian target of rapamycin (mTOR) by phosphorylating Ser2448 and tuberous sclerosis complex 2 (TSC2) to activate mTORC1 [3]. Cells have evolved multiple mechanisms to maintain DNA fidelity in response to DNA damage, such as double-strand breaks (DSBs). PARP-1, a member of the poly(ADP-ribose) (PAR) polymerase family, exhibits robust DNA damage repair capacity through its PARylation activity [4].

PARP inhibitors bind to PARP1, inhibiting its enzymatic activity and leading to the accumulation of irreparable single-strand breaks, which eventually trigger double-strand breaks (DSBs) and initiate homologous recombination (HR) repair [5]. PARP inhibitors (PARPi) have been widely used in cancer treatment, exploiting the “synthetic lethality” phenomenon in HRD cancers by preventing HR-mediated repair, thereby promoting cancer cell apoptosis. However, tumors eventually develop resistance to PARPi [6,7]. Inhibition of the PI3K-AKT-mTOR pathway can delay cancer progression; combining the PI3K-AKT-mTOR inhibitor BKM120 with PARPi in ovarian cancer cells yields better tumor suppression than PARPi alone, providing a novel strategy for treating drug-resistant cancer cells and improving patient prognosis [8,9].

MicroRNAs (miRNAs) are short RNA molecules that regulate gene expression through post-transcriptional silencing of target genes, participating in the pathogenesis of various diseases, including allergic, neurodegenerative, cardiac, and fibrotic disorders [10]. MiRNAs play a key role in cell proliferation and development [11,12,13,14,15,16]. Subtypes of the miR-320 family have been implicated in cancer initiation and progression [17], whereas miR-320e uniquely functions in the pathogenesis of cardiometabolic disorders and cognitive dysfunction [18,19].

The specific objective of this study was to investigate the regulatory role of miR-320e in ovarian cancer progression and its potential involvement in the response to PARPi treatment. Our results demonstrate that miR-320e inhibits ovarian cancer cell proliferation and promotes apoptosis and autophagy by regulating the PI3K-AKT-mTOR signaling pathway. Furthermore, combining miR-320e with olaparib significantly reduces ovarian cancer resistance to PARPi treatment. These findings provide experimental evidence for exploring the potential regulatory role of the miR-320e-PI3K-AKT-mTOR axis in PARPi response in ovarian cancer.

## 2 Materials and Methods

### 2.1 Cell Cultures of Human Ovarian Cancer Cells

A2780 (CM-0013), SKOV3 (CL-0215), 293T (CM-0005) and HEK293 (CL-0001) cell lines were all purchased from Procell Life Science & Technology Co., Ltd. (Wuhan, China). All cell lines were authenticated within the past three years: genomic DNA was extracted from A2780 and SKOV3 cells using the TINAmp Genomic DNA Kit (Cat. No. DP304, TIANGEN Biotech Co., Ltd., Beijing, China); 20 STR loci and gender identification loci were amplified with the Microreader™ 21 ID system (Cat. No. MR-21ID, Beijing Microread Genetics Co., Ltd., Beijing, China), and PCR products were analyzed via a GenReader 7010 Gene Analyzer (Applied Biosystems, Foster City, CA, USA) equipped with GeneMapper Software 6.0 (Applied Biosystems), followed by comparison against the ExPASy database (ExPASy Proteomics Server, Swiss Institute of Bioinformatics, Geneva, Switzerland). STR typing was performed using the aforementioned GenReader 7010 Gene Analyzer and GeneMapper Software 6.0 (Applied Biosystems), which confirmed no cross-contamination in either cell line. All cells were tested and confirmed to be free of mycoplasma contamination. Cells were cultured in RPMI 1640 medium (Cat. No. 11875093, Gibco, Thermo Fisher Scientific, Grand Island, New York, USA) supplemented with 10% fetal bovine serum (FBS, Cat. No. 10091148, Gibco) and 1% penicillin-streptomycin solution (Cat. No. P1400, Solarbio, Beijing, China) in a humidified 37°C incubator with 5% CO_2_.

### 2.2 Cell Transfection

MiR-320e mimics (5′-AAAGCUGGGUUGAGAAGG-3′) and their negative control (mimics-NC, 5′-UUCUCCGAACGUGUCACGUTT-3′) were purchased from RiboBio Co., Ltd. (Guangzhou, Guangdong, China). A2780 and SKOV3 were seeded in 6-well plates at a density of 2 × 10^5^ cells/well and allowed to adhere for at least 12 h. Transfection was performed using the riboFECT CP Transfection Kit (Cat. No. C10511-05, RiboBio). Briefly, 5 μL of 20 μM siRNA stock solution was added to 120 μL of 1× riboFECT™ CP Buffer, followed by gentle mixing. Then, 12 μL of riboFECT™ CP Reagent was added, and the mixture was gently pipetted up and down before incubation at room temperature for 0–15 min to prepare the transfection complex. The complex was added to cells cultured in serum-free medium. At 24 h post-transfection, the serum-free medium was replaced with serum-supplemented complete medium for continuous culture. All experiments were performed in three independent biological replicates, each with three technical replicates.

### 2.3 Fluorescence In Situ Hybridization (FISH)

First, we measured miR-320e expression in 20 patients with high-grade serous ovarian cancer (HGSOC) and 20 patients with benign ovarian lesions. Biotin-labeled RNA probes specific to miR-320e were transcribed from PCR fragments using a biotin-labeling mix and RNA polymerase, following the manufacturer’s instructions (hsa-miR-320e, probe:5′-CCTTCTCAACCCAGCTTT-3′) (Cat. No. 7605130001, Roche Diagnostics, Indianapolis, Indiana, USA). Prior to hybridization, slides were subjected to a series of prehybridization procedures. Briefly, paraffin sections were dewaxed completely and rehydrated through a graded ethanol series. Antigen retrieval was then performed under high temperature and pressure conditions. Subsequently, tissue sections were incubated with pepsin/protease solution for controlled protease digestion to expose target nucleic acid fragments. After rinsing, the sections were treated with blocking solution to eliminate non-specific binding background before subsequent FISH hybridization. Ovarian tissues were sectioned at 4 μm, fixed in formalin, and subsequently hybridized with the miR-320e-specific biotin-labeled probes in hybridization buffer. Signals were detected using a tyramide-conjugated Alexa Fluor^®^ 488 fluorochrome TSA kit (Cat. No. T20912, Thermo Fisher Scientific). Images were acquired with a laser scanning confocal microscope (LSM 880, Carl Zeiss, Oberkochen, Baden-Württemberg, Germany) at 400× magnification. Fluorescence signals were quantitatively analyzed using ImageJ software. ROIs were manually delineated to remove non-specific background. For each sample, at least three random visual fields (≥50 cells per field) were selected. The integrated optical density (IOD) and area of FISH-positive miRNA signals were determined, and the mean fluorescence intensity (MFI) was calculated as IOD/ROI area to evaluate the relative expression of target miRNA. All experiments were performed in three independent biological replicates.

### 2.4 CCK8 Assay

A2780 and SKOV3 were seeded in 96-well plates at a density of 2 × 10^3^ cells/well and cultured in RPMI 1640 medium (Cat. No. 11875093, Gibco) containing 10% FBS (Cat. No. 10091148, Gibco). Following 24-h transfection, cells were cultured for various durations (24, 48, 72, 96, and 120 h). The RPMI 1640 medium (Cat. No. 11875093, Gibco) was replaced at 72 and 120 h. At each time point, 10 μL of CCK-8 reagent (Cat. No. KGA1606-500, KeyGEN BioTECH, Nanjing, Jiangsu, China) was added to each well. After incubation for 1 h at 37°C with 5% CO_2_, cell proliferation was assessed by measuring the optical density using a BIO-RAD microplate reader (Cat. No. 168-1130; Bio-Rad Laboratories, Inc., Hercules, California, USA) at 450 nm. All experiments were performed in three independent biological replicates, each with three technical replicates.

### 2.5 Wound Healing Assay

A2780 and SKOV3 (1 × 10^5^) were seeded in 6-well plates and cultured until reaching 90% confluence. At 24 h post-transfection, a 200 μL pipette tip was used to create a scratch wound in the confluent cell monolayer. Plates were gently washed three times with PBS to remove detached cells, then refreshed with serum-free RPMI 1640 medium (Cat. No. 11875093, Gibco). Wound closure was observed and imaged at 0 h (immediately after scratching) and 24 h using a Olympus BX53 upright microscope (Olympus Corp, Hachioji-shi, Tokyo, Japan). The wound healing area was quantified using ImageJ software (v1.54p, National Institutes of Health, Bethesda, Maryland, USA). All experiments were performed in three independent biological replicates, each with three technical replicates.

### 2.6 Transwell Assay

A2780 and SKOV3 (4 × 10^4^) were suspended in serum-free RPMI 1640 medium (Cat. No. 11875093, Gibco) and seeded into the upper inserts of 6.5 mm Transwell chambers (8.0 μm pore polycarbonate membrane, Cat. No. 3422; Corning Inc., Corning, New York, USA). For the migration assay, the lower plates contained RPMI 1640 medium (Cat. No. 11875093, Gibco) supplemented with 10% FBS (Cat. No. 10091148, Gibco) as a chemoattractant. For the invasion assay, the upper inserts were pre-coated with 50 μL of Matrigel (Cat. No. 356234, Corning Inc., USA) diluted 1:8 with serum-free RPMI 1640 medium (Cat. No. 11875093, Gibco), and the Matrigel was incubated at 37°C for 30–45 min to form a solid gel layer before seeding the cells; the lower plates were prepared identically to the migration assay with RPMI 1640 medium (Cat. No. 11875093, Gibco) supplemented with FBS (Cat. No. 10091148, Gibco) as a chemoattractant. After incubation for 24 h at 37°C in a humidified 5% CO_2_ atmosphere, the inserts were removed, and the upper surface of the membrane was scrubbed to eliminate non-migratory or non-invasive cells. The Transwell inserts were fixed with 4% paraformaldehyde for 15–20 min, followed by PBS washing and staining with 0.1% crystal violet at room temperature for 15 min. The non-migrated or non-invaded cells on the upper membrane surface were carefully removed with a cotton swab. Migrated cells (for migration assay) and invaded cells (for invasion assay) on the lower surface were imaged under a microscope, and the average number of migrated or invaded cells in each group was quantified. All experiments were performed in three independent biological replicates, each with three technical replicates.

### 2.7 EdU Assay

Cell proliferation was detected using the EdU incorporation assay. Briefly, A2780 and SKOV3 cells were seeded and cultured overnight. After treatment, cells were incubated with EdU kit (Cat. No. C10310-1, RiboBio) at 37°C for 2 h. Subsequently, cells were fixed with 4% paraformaldehyde for 15–20 min and permeabilized with 0.5% Triton X-100 for 10 min at room temperature. Then, Apollo staining was performed in the dark for 30 min, followed by nuclear counterstaining with Hoechst 33342 for 10 min. Results were observed and imaged with a fluorescence microscope (DM2500, Leica Microsystems, Wetzlar, Hesse, Germany). All experiments were performed in three independent biological replicates, each with three technical replicates.

### 2.8 Colony Formation Assay

Colony formation assay was used to assess the clonogenic capacity of A2780 and SKOV3 cells. Briefly, the two cell lines were trypsinized into single-cell suspensions and seeded in 6-well plates at 100–200 cells/well. Cells were cultured in RPMI-1640 medium (Cat. No. 11875093, Gibco) with 10% FBS (Cat. No. 10091148, Gibco) at 37°C, 5% CO_2_, with medium refreshed every 3 days for 14 days until visible colonies formed. After culture, medium was discarded, and cells were gently washed twice with PBS. Cells were fixed with 4% paraformaldehyde at room temperature for 15–20 min, washed three times with PBS (5 min each), then stained with 0.1% crystal violet in the dark for 15 min. After staining, excess dye was rinsed off with tap water until colony edges were clear. Plates were air-dried, colonies (≥50 cells/cluster) were imaged under an BX53 Olympus microscope, and colony number was manually counted to calculate the colony formation rate. All experiments were performed in three independent biological replicates, each with three technical replicates.

### 2.9 Measurement of Cell Apoptosis

A2780 and SKOV3 cells apoptosis rates were detected using an Annexin V-fluorescein isothiocyanate (FITC)/propidium iodide (PI) apoptosis kit (Cat. No. CA1020, Solarbio). Cells were seeded in 6-well plates at 2 × 10^5^ to 5 × 10^5^ cells per well and cultured for 24 h, followed by 48 h of treatment with one of the following: miR-320e mimic, mimic negative control (mimic-NC), olaparib, or a combination of miR-320e mimic/mimic-NC with olaparib. For each sample, 5 μL of Annexin V-FITC and 5 μL of PI were added sequentially, and the total staining volume was adjusted to 100 μL with binding buffer. After 15 min of incubation at room temperature in the dark, samples were analyzed using a BD FACSVerse™ flow cytometer (BD Biosciences, San Jose, CA, USA).

In the flow cytometric dot plot, viable cells were defined as Annexin V-FITC^−^/PI^−^, early apoptotic cells as Annexin V-FITC^+^/PI^−^, late apoptotic/necrotic cells as Annexin V-FITC^+^/PI^+^, and necrotic cells as Annexin V-FITC^−^/PI^+^. The total apoptotic rate was calculated as the sum of early apoptotic and late apoptotic cell percentages. All experiments were performed in three independent biological replicates, each with three technical replicates.

### 2.10 Immunofluorescence Assays

A2780 and SKOV3 cells were seeded into 6-well plates at 2 × 10^5^ cells/well and cultured at 37°C with 5% CO_2_. When cell confluency reached 60%–70%, they were divided into four groups: (1) miR-320e transfection group (20 μM, 48 h transfection); (2) blank transfection control group (20 μM mimic-NC, 48 h transfection); (3) Olaparib + miR-320e group (20 μM miR-320e transfection for 48 h, followed by olaparib treatment for 24 h); (4) Olaparib + blank transfection group (20 μM mimic-NC transfection for 48 h, followed by olaparib treatment for 24 h).

After transfection and drug treatment, immunofluorescence staining was performed. Cells were washed with pre-cooled PBS, fixed with 4% paraformaldehyde, permeabilized with 0.1% Triton X-100, and blocked by incubating cells with 5% bovine serum albumin (BSA, Cat. No. ST023, Beyotime Biotechnology, Shanghai, China) for 1 h at room temperature, followed by overnight incubation at 4°C with primary antibodies: anti-γ-H2AX (Cat. No. 2577S, Cell Signaling Technology, Inc., Danvers, MA, USA) and anti-RAD51 (Cat. No. 65653S, Cell Signaling Technology), both diluted 1:200. After three further PBS washes, samples were incubated with Alexa Fluor^®^ 488- and 563-conjugated secondary antibodies (Cat. No. P0188-1 and Cat. No. A0473, Beyotime Biotechnology, Inc., Songjiang District, Shanghai, China), each diluted 1:1000, for 1 h at room temperature. Finally, cells were stained with 4′,6-diamidino-2-phenylindole (DAPI) (Cat. No. C1002, Beyotime Biotechnology) for 15 min at room temperature. Images were captured using a DM2500 fluorescence microscope (Leica). All experiments were performed in three independent biological replicates, each with three technical replicates.

### 2.11 RNA Isolation and qRT-PCR

RNA purity was assessed via OD260/280 and OD260/230 absorbance ratios. Samples with OD260/280 between 1.8–2.0 and OD260/230 above 2.0 were regarded as qualified. Exactly 1 μg of total RNA was applied for reverse transcription. Total RNA was extracted from A2780 and SKOV3 cells using TRIzol reagent (Cat. No. 15596026, Thermo Fisher Scientific). For the reverse transcription of total RNA to cDNA, the FastKing RT Kit (with gDNase) (Cat. No. KR116-03, Tiangen Biotech, Beijing, China) was used to effectively remove genomic DNA contamination and ensure the purity of cDNA. The qPCR reaction was performed in a total volume of 20 μL, containing 10 μL of SuperReal PreMix Plus (SYBR Green) (Cat. No. FP205-02, Tiangen Biotech) for detection, 2 μL of cDNA template, and 7 μL of nuclease-free water. The cycling conditions were set as follows: initial denaturation at 95°C for 20 s, followed by 40 cycles of denaturation at 95°C for 1 s and annealing/extension at 60°C for 20 s. After the amplification cycles, a melting curve analysis was conducted from 60°C to 95°C (increasing by 0.5°C every 5 s) to confirm the specificity of the PCR products on a 7500 Real-time PCR System (Cat. No. 4351108 Applied Biosystems). GAPDH was selected as the internal reference gene for sample normalization. Relative mRNA expression levels were calculated adopting the 2^−ΔΔCt^ method. All experiments were performed in three independent biological replicates, each with three technical replicates.

The primers used were as follows:

FN1 forward: 5′-ACAGAACTATGATGCCGACCAGAAG-3′
FN1 reverse: 5′-CTGATCTCCAATGCGGTACATGA-3′
GAPDH forward: 5′-GGAGCGAGATCCCTCCAAAAT-3′
GAPDH reverse: 5′-GGCTGTTGTCATACTTCTCATGG-3′
hsa-miR-320e forward primer: 5′-GGCAGTGTCTTAGTGGGGTT-3′
hsa-miR-320e reverse primer: 5′-CAGTGCAGGGTCCGAGGT-3′
U6 snRNA forward primer: 5′-CTCGCTTCGGCAGCACATATACT-3′
U6 snRNA reverse primer: 5′-ACGCTTCACGAATTTGCGTGTC-3′.

### 2.12 Dual Luciferase Reporter Assay

Binding sites between miR-320e and FN1 were predicted using the bioinformatics tool TargetScan Human 8.0. The fragment of the FN1 3′-UTR containing either the predicted miR-320e binding sequence or its mutant variant was synthesized and cloned into the pmirGLO vector (Cat. No. HS-10001, HeSheng, Beijing, China). Human HEK293 cells (CL-0001, Procell Life Science & Technology Co., Ltd.) were co-transfected with the luciferase reporter vector, either alone or in combination with the miR-320e mimic. Cells were cultured for 36 h under standard incubation conditions (37°C, 5% CO_2_). Luciferase activity was then determined by the Dual-Luciferase Reporter Assay System (Cat. No. E1910, Promega Corporation, Madison, Wisconsin, USA) following the manufacturer’s protocol, with results normalized to Renilla luciferase activity. All experiments were performed in three independent biological replicates, each with three technical replicates.

### 2.13 Western Blot (WB) Analysis

Samples including transfected and drug-treated A2780, SKOV3 cells as well as mouse tumor tissues were lysed in RIPA buffer supplemented with protease inhibitors (P1006, Beyotime Biotechnology) at 4°C. Protein concentration was determined using a BCA Protein Assay Kit (Cat. No. 23225, Thermo Fisher Scientific). A total of 50 μg of protein per sample was separated by 8%–10% SDS-PAGE and transferred onto a 0.2 μm pore-size PVDF membrane. Information for antibodies against related proteins is provided in Table S1: Antibody Information for Western Blot. After blocking with 5% non-fat dried milk in TBST at room temperature for 2 h, the membrane was incubated with primary antibodies overnight at 4°C. After 2 h of incubation with HRP-conjugated secondary antibodies (in Table S1) at room temperature, HRP signals were detected using a hypersensitive ECL Chemiluminescence Detection Kit (Cat. No. WBKLS0100, Merck KGaA, Darmstadt, Germany). Blot images were analyzed via densitometry using Quantity One v4.6.2 software (Bio-Rad, CA, USA). GAPDH served as the loading control. All experiments were performed in three independent biological replicates, each with three technical replicates.

### 2.14 Xenograft Tumor Growth Study

A total of 80 female BALB/c nude mice (6–8 weeks of age, body weight 18–22 g) were purchased from Shulaibao Biotechnology Co., Ltd. (Wuhan, China). The animal experimental protocol in this study was reviewed and approved by the Animal Ethics Committee of Sino Animal (Beijing) Science and Technology Development Co., Ltd. (Approval No.: 20230048YZH-3R; Approval Date: 02 March 2023), and strictly performed in accordance with the ARRIVE Essential 10 guidelines and institutional animal welfare regulations.

Mice were housed under specific pathogen-free (SPF) conditions with a 12 h light/dark cycle, constant temperature (22 ± 2°C) and humidity (50%–60%), with free access to standard laboratory chow and sterile drinking water. After one week of acclimatization, subcutaneous ovarian cancer xenograft models were established by injecting 5 × 10^6^ A2780 cells suspended in 100 μL sterile PBS into the axillary region of each nude mouse.

Tumor length and width were measured every 3 days using a digital caliper, and tumor volume was calculated according to the formula: Tumor volume (mm^3^) = length × width^2^/2. Mice that failed to develop detectable subcutaneous tumors were excluded prior to randomization. The remaining tumor-bearing mice were randomly allocated into four experimental groups, with eight mice per group:

(1) Intratumoral injection of micrON agomir negative control (agomir-NC);
(2) Intratumoral injection of micrON mmu-miR-320e agomir;
(3) Intraperitoneal injection of olaparib combined with intratumoral injection of agomir-NC;
(4) Intraperitoneal injection of olaparib combined with intratumoral injection of micrON mmu-miR-320e agomir.

When the average xenograft volume reached approximately 100 mm^3^, olaparib was administered via intraperitoneal injection at a dose of 3 mg/kg every 3 days. Three days after the first olaparib administration, intratumoral injection of mmu-miR-320e agomir or agomir-NC was performed every 3 days at a fixed dose of 3 nmol per mouse.

Humane endpoint criteria were predefined as follows: maximum tumor diameter reaching 20 mm, tumor volume exceeding 1000 mm^3^, apparent mental distress, body weight loss over 20%, tumor ulceration, or impaired locomotor activity. Mice meeting any humane endpoint criterion were humanely euthanized. At the experimental endpoint, all mice were euthanized, and xenograft tissues were dissected, weighed, and preserved for subsequent molecular and histological analyses. All *in vivo* experiments were conducted in three independent biological replicates.

### 2.15 Immunohistochemistry

Tissue samples were collected from 20 patients with benign ovarian lesions and 20 patients with high-grade serous ovarian cancer (HGSOC) at the Department of Obstetrics and Gynecology, Beijing Chao-Yang Hospital, Capital Medical University. Tissues were fixed in 10% formaldehyde solution, embedded in paraffin, and sectioned at 4 μm thickness. Briefly, sections were deparaffinized, blocked with 3% hydrogen peroxide, and subjected to antigen retrieval by pressure cooking in 10 mM citrate buffer (pH 6.0). After incubation with primary antibodies (in Table S1) for 1 h, sections were incubated with HRP-conjugated anti-mouse/rabbit IgG (Cat. No. SA00001, Beyotime Biotechnology) was incubated at a dilution of 1:5000 for 30 min at room temperature in a humidified chamber. Antibody binding was visualized using 3,3′-diaminobenzidine (Cat. No. P0202, Beyotime Biotechnology). Immunohistochemical images were captured using an Olympus BX53 microscope (Olympus Corp, Japan). The positive staining area and relative expression level were quantitatively analyzed by ImageJ software (v1.54p, National Institutes of Health, Bethesda, MD, USA). Staining results were evaluated by the H-score method, and all assessments were conducted under blinded conditions. All experiments were performed in three independent biological replicates, each with three technical replicates.

### 2.16 Transcriptome Sequencing and Bioinformatics Analysis

Human ovarian cancer A2780 cells were divided into two experimental groups: mimic-NC group (transfected with miR-320e negative control mimic) and miR-320e mimic group (transfected with miR-320e mimic). After routine cell culture and logarithmic growth phase synchronization, cell transfection was performed according to the manufacturer’s protocol. At 48 h post-transfection, adherent cells were collected, and total RNA was extracted using TRIzol reagent (Cat. No. 15596026, Invitrogen). RNA integrity, concentration and purity were assessed by agarose gel electrophoresis, Nanodrop spectrophotometer and Agilent 2100 Bioanalyzer to ensure qualified RNA samples for library construction.

RNA-seq library preparation and high-throughput sequencing were performed on the Illumina NovaSeq platform. Raw reads were filtered to remove adapter sequences, low-quality reads and ambiguous bases to obtain clean reads. The clean reads were aligned to the human reference genome using HISAT2 software. Gene expression levels were quantified and normalized to FPKM/TPM values.

Differentially expressed genes (DEGs) between the miR-320e mimic group and mimic-NC group were screened with the thresholds of |log_2_ FC| ≥ 1 and adjusted *p*-value < 0.05. Gene Ontology (GO) functional enrichment and Kyoto Encyclopedia of Genes and Genomes (KEGG) pathway enrichment analysis were subsequently performed for DEGs. Protein–protein interaction (PPI) network was constructed using the STRING database, and hub genes were identified by network topology analysis. All bioinformatics analyses were conducted using R software and related bioinformatics packages.

### 2.17 Statistical Analysis

All statistical analyses were performed using GraphPad Prism software (v9.3, GraphPad Software Inc., San Diego, California, USA). FlowJo software v11.1.1 (BD Biosciences, Ashland, OR, USA) was used to calculate the apoptosis rate from flow cytometry data. ImageJ software (v1.54p, National Institutes of Health, USA) was applied to quantify the gray intensity of Western blot (WB) results.

Data are presented as the mean ± standard deviation (SD) from at least three independent experiments. For comparisons between two groups, unpaired Student’s *t*-test or Mann–Whitney U test was used. For three or more groups, one-way ANOVA was performed, followed by Tukey HSD post hoc test for multiple comparisons. A *p*-value < 0.05 was considered statistically significant.

### 2.18 Ethics Approval and Consent to Participate

#### 2.18.1 Human Research Ethics Statement

This study involving human participants was conducted after a detailed explanation of the research content. All procedures complied with the ethical principles outlined in the Declaration of Helsinki. The human study protocol was approved by the Human Research Ethics Committee of Beijing Chao-Yang Hospital, Capital Medical University (Approval No.: 2019-Tec-66).

#### 2.18.2 Animal Experimental Ethics Statement

All animal surgical operations were performed under sodium pentobarbital anesthesia, and all necessary measures were taken to minimize animal suffering and distress. The animal experimental protocol in this study was reviewed and approved by the Animal Ethics Committee of Sino Animal (BeiJing) Science and Technology Development Co., Ltd. (Approval No.: 20230048YZH-3R; Approval Date: March 2, 2023). All animal experiments were carried out in accordance with institutional and national guidelines for the care and use of laboratory animals.

## 3 Results

### 3.1 miR-320e Reduces Proliferation and Migration but Promotes Apoptosis in Human Ovarian Cancer Cell Lines

**Figure 1:**
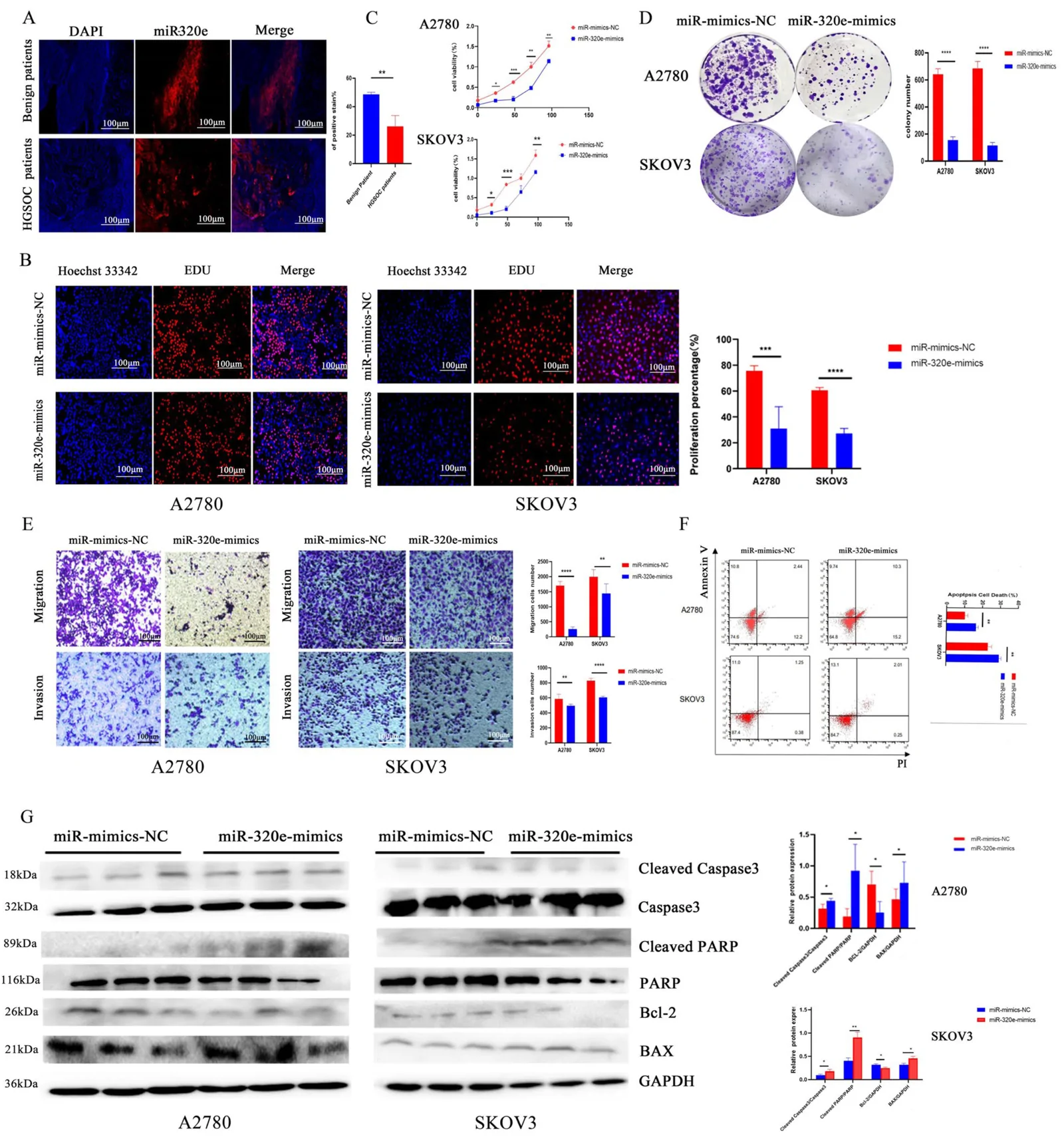
**MiR-320e tissue expression and biological function.** (**A**) The expression level of miR-320e in benign ovarian cancer tissues measured by FISH experiment was significantly higher than that in HGSOC patients. Compared with the control group, overexpression of miR-320e in A2780 (**B**) and SKOV3 (**C**) cells resulted in a significant decrease in their proliferation ability. (**D**) Compared with the control group, overexpression of miR-320e resulted in a significant decrease in the number of clonal clusters formed by both types of cells. (**E**) Compared with the control group, overexpression of miR-320e significantly reduced the migration and invasion ability of both types of cells. (**F**) The flow cytometry results showed that overexpression of miR-320e significantly increased the proportion of apoptosis in both types of cells. (**G**) The WB experiment results showed that overexpression of miR-320e in A2780 and SKOV3 cells resulted in increased expression of cleaved caspase3/caspase3 and cleaved PARP/PARP and BAX, while decreased expression of Bcl-2. Biological replicates (n = 3). *p < 0.05, **p < 0.01, ***p < 0.001, ****p < 0.0001.

Initially, we conducted FISH experiments to measure the expression of miR-320e in human ovarian cancer tissues. The results showed that the positive region of miR-320e was significantly larger in benign patients compared to HGSOC patients (Fig. 1A). The transfection efficiency of miR-320e in the two types of cells is shown in Fig. 1: The Transfection Efficiency. To verify the effect of miR-320e on cell proliferation and migration in ovarian cancer cells, we measured their capacity for proliferation and migration. EdU experiments revealed a decrease in the cell proliferation ratio after transfection with miR-320e in both cell types (Fig. 1B). Compared to the control group, transfection with miR-320e significantly decreased the proliferation ability of A2780 and SKOV3 cells as determined by CCK8 assay (Fig. 1C). Colony formation experiments demonstrated a significant reduction in colony formation after transfection with miR-320e (Fig. 1D). Migration and invasion assays showed that transfection with miR-320e significantly inhibited the migration and invasion ability of A2780 and SKOV3 cells (Fig. 1E). Flow cytometry results showed that transfection with miR-320e significantly increased the apoptosis ratio in both cell types compared to the control group (Fig. 1F). WB experiments revealed an increase in the expression ratios of cleaved caspase3/caspase3 and cleaved PARP/PARP, indicating activation of the apoptotic pathway in the experimental group. Furthermore, the decrease in anti-apoptotic protein Bcl-2 expression and increase in pro-apoptotic protein BAX confirmed apoptosis activation (Fig. 1G).

### 3.2 Transfection of miR-320e Inhibits the PI3K-AKT-mTOR Signaling Pathway and Activates Autophagy in Ovarian Cancer Cells

**Figure 2:**
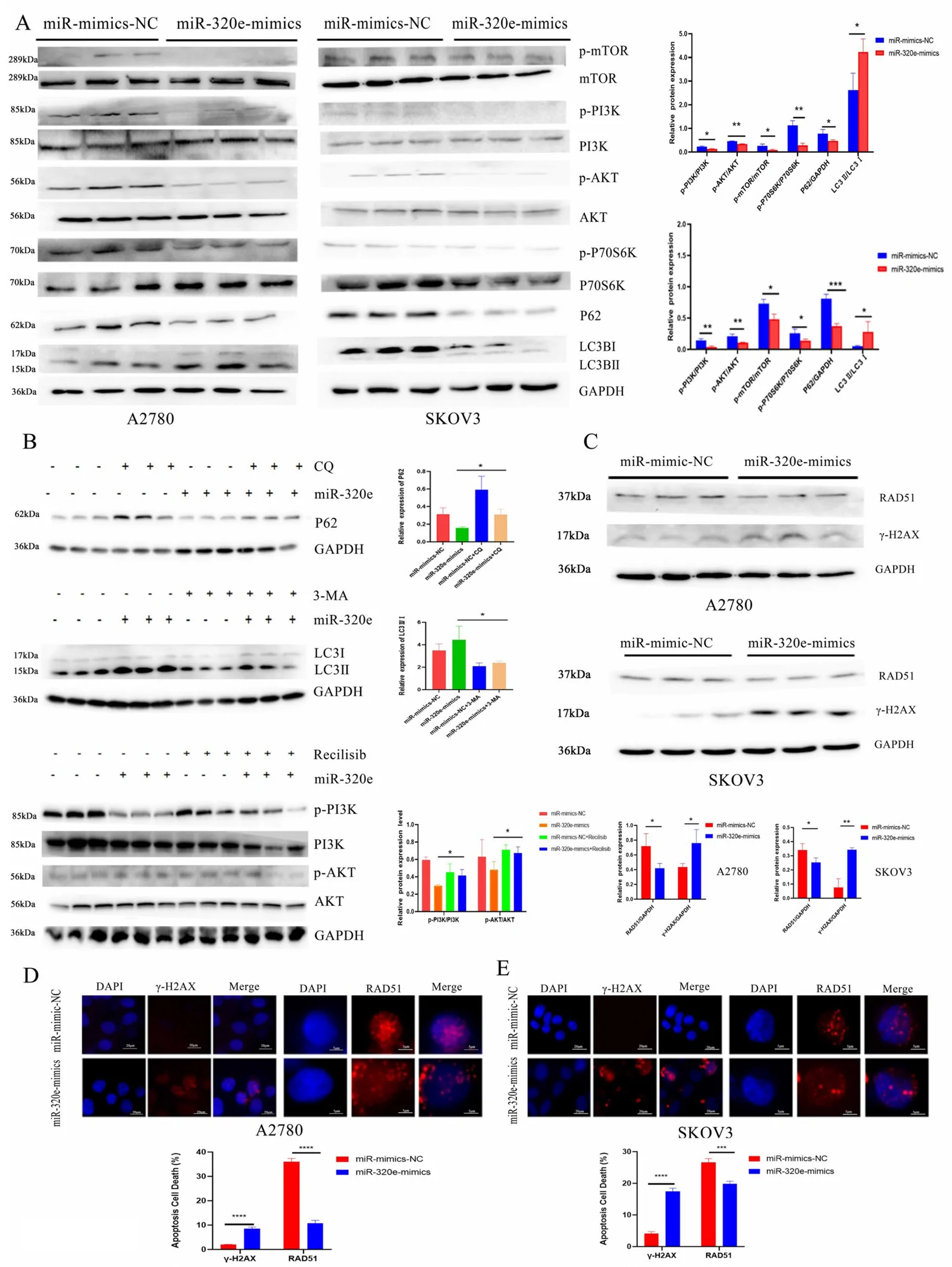
**MiR-320e regulates PI3K-AKT-mTOR pathway, autophagy and apoptosis.** (**A**) miR-320e inhibits the activation of the PI3K-AKT-mTOR signaling pathway in ovarian cancer cells and promotes autophagy and apoptosis in ovarian cancer cells. (**B**) The effect of miR-320e on decreasing P62 expression can be restored by CQ; the effect of miR-320 on increasing the ratio of LC3II/LC3I can be restored by 3-MA; the inhibitory effect of miR-320 on the PI3K-AKT-mTOR pathway can be restored by Recilisib. (**C**) WB assay showed that miR-320e promotes the expression of γ-H2AX and in the nucleus of A2780 and SKOV3 cells. Immunofluorescence experiments had shown that miR-320e decreased the expression of RAD51 in the nucleus of A2780 (**D**) and SKOV3 (**E**) cell. Biological replicates (n = 3). *p < 0.05, **p < 0.01, ***p < 0.001, ****p < 0.0001.

Based on our experimental results, we predict that miR-320e plays a role in regulating the PI3K-AKT-mTOR signaling pathway in ovarian cells. WB results showed that compared to the control group, the treatment group had decreased expression of phosphorylated (p)-PI3K/PI3K, (p)-AKT/AKT, (p)-mTOR/mTOR, and p-p70s6k/p70s6k (Fig. 2A). Studies have shown that changes in the PI3K-AKT-mTOR signaling pathway can cause corresponding changes in the autophagy process [20]. We further examined autophagy markers P62 and LC3B and found a significantly upregulated ratio of LC3BII/LC3BI and decreased expression of P62 (Fig. 2A). Furthermore, following intervention with Recilisib (Cat. No. E2947, Selleck Chemicals, Houston, Texas, USA), 3-MA (Cat. No. A8780, Solarbio) and CQ (Cat. No. Y264393, Beyotime Biotechnology), the reduced activity of the PI3K-AKT-mTOR pathway and elevated autophagy caused by miR-320e transfection showed a numerical increase, whereas the overall biological trend was not reversed (all *p* < 0.05; Fig. 2B). These results suggest that transfection of miR-320e can inhibit the PI3K-AKT-mTOR signaling pathway and activate downstream autophagy in A2780 and SKOV3 cells.

### 3.3 Transfection of miR-320e Induces DNA Damage in Ovarian Cancer Cells

As mentioned earlier, inhibiting the PI3K-AKT-mTOR pathway can induce DNA damage in ovarian cancer cells with HRD ^9^, thereby enhancing PARPi’s intracellular effects. This process promotes apoptosis and inhibits cancer cell growth. Therefore, we hypothesized that while inhibiting this pathway, miR-320e may also promote DNA damage. WB assay showed an increase in γ-H2AX levels (a marker for early DNA double-strand breaks) after miR-320e transfection in both A2780 and SKOV3 cells (all *p* < 0.05; Fig. 2C). In contrast, there was a decrease in RAD51 positivity (a marker for damaged DNA repair) (all *p* < 0.05; Fig. 2C). The immunofluorescence assay results for γ-H2AX and RAD51 were consistent with those from the WB assay (all *p* < 0.0001; Fig. 2D; all *p* < 0.001; Fig. 2E).

### 3.4 Overexpression of miR-320e Enhances the Sensitivity of Ovarian Cancer Cells to Olaparib Therapy

**Figure 3:**
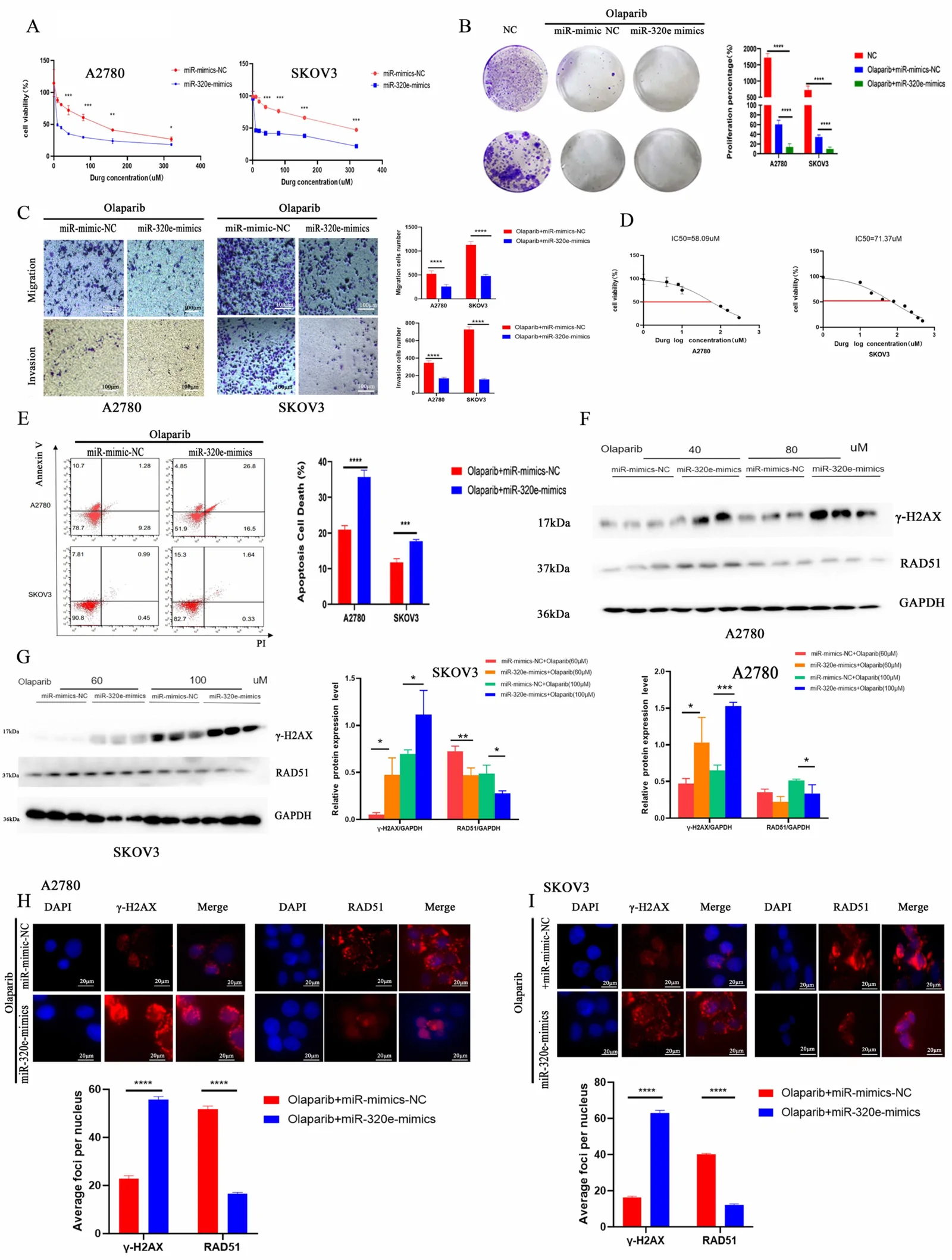
**MiR-320e increases ovarian cancer cell sensitivity.** (**A**) Cells to olaparib CCK8 experiment showed that under different concentrations of olaparib (0-400 μM), compared with the control group, the proliferation rate of A2780 and SKOV3 cells in the overexpressed miR-320e group was significantly reduced. (**B**) Compared with the olaparib treatment group, the number of clones formed by ovarian cancer cells after overexpression of miR-320e was significantly reduced. (**C**) The results of Transwells experiment showed that the overexpression of miR-320e significantly enhanced the inhibitory effect of olaparib on the migration and invasion of A2780 and SKOV3. (**D**) The IC50 values of A2780 and SKOV3 under olaparib were 58.09 μM and 71.37 μM. (**E**) Flow cytometry results showed that overexpression of miR-320e in two types of ovarian cancer cells significantly increased the proportion of apoptosis induced by olaparib treatment. WB experiment results showed that overexpression of miR-320e could significantly enhance the increase of γ-H2AX expression and the decrease of RAD51 expression in A2780 (**F**) and SKOV3 (**G**) induced by olaparib treatment. Immunofluorescence assay results showed that overexpression of miR-320e could significantly enhance the increase of γ-H2AX expression and the decrease of RAD51 expression in the nucleus of two cancer cells induced by olaparib treatment (**H**,**I**). Biological replicates (n = 3). *p < 0.05, **p < 0.01, ***p < 0.001, ****p < 0.0001.

We conducted a series of experiments using different concentrations of olaparib (0–400 μM) to compare survival rates between control and treatment groups after drug stimulation. Based on the results from the CCK8 experiment, we observed a significant reduction in cell survival rate in the experimental group compared to the control group after olaparib treatment (all *p* < 0.05; Fig. 3A). The colony formation experiment showed that after olaparib treatment, the number of cell colonies in the treated group was significantly reduced compared to the control group, and transfection with miR-320e significantly reduced the number of colonies compared to the control group treated with olaparib (*p* < 0.0001; Fig. 3B). The Transwell assay demonstrated that after olaparib treatment, the migration and invasion ability of the treated group was significantly reduced compared to the control group, and transfection with miR-320e significantly induced a lower number of migrating and invading cells in the treatment group compared to the control group treated with olaparib (*p* < 0.0001; Fig. 3C). These findings indicate that miR-320e promotes sensitivity to olaparib in ovarian cancer cells while inhibiting their proliferation, invasion, and migration abilities.

To determine the appropriate concentration of olaparib, we used the CCK8 assay to calculate the IC50 values for A2780 and SKOV3 cells as 58.09 μM and 71.37 μM, respectively (Fig. 3D). Flow cytometry analysis revealed that the apoptosis rates were higher in the miR-320e overexpression group with olaparib treatment than in the control group with olaparib treatment (*p* < 0.001; Fig. 3E). We selected two concentrations near the IC50 values as the action concentrations: 40 μM and 80 μM for A2780 cells, and 60 μM and 100 μM for SKOV3 cells. WB experiments (all *p* < 0.05; Fig. 3F,G) and immunofluorescence experiments showed a significant upregulation of γ-H2AX expression in the treatment group, while RAD51 expression was downregulated, indicating that transfection with miR-320e mimics enhances DNA damage caused by olaparib in ovarian cancer cells (*p* < 0.0001; Fig. 3H; *p* < 0.0001; Fig. 3I). These results suggested that miR-320e can enhance sensitivity to olaparib in both A2780 and SKOV3 cell lines.

### 3.5 miR-320e Enhances Ovarian Cancer Cell Sensitivity to Olaparib by Inhibiting the PI3K-AKT-mTOR Pathway and Activating Autophagy and Apoptosis

**Figure 4:**
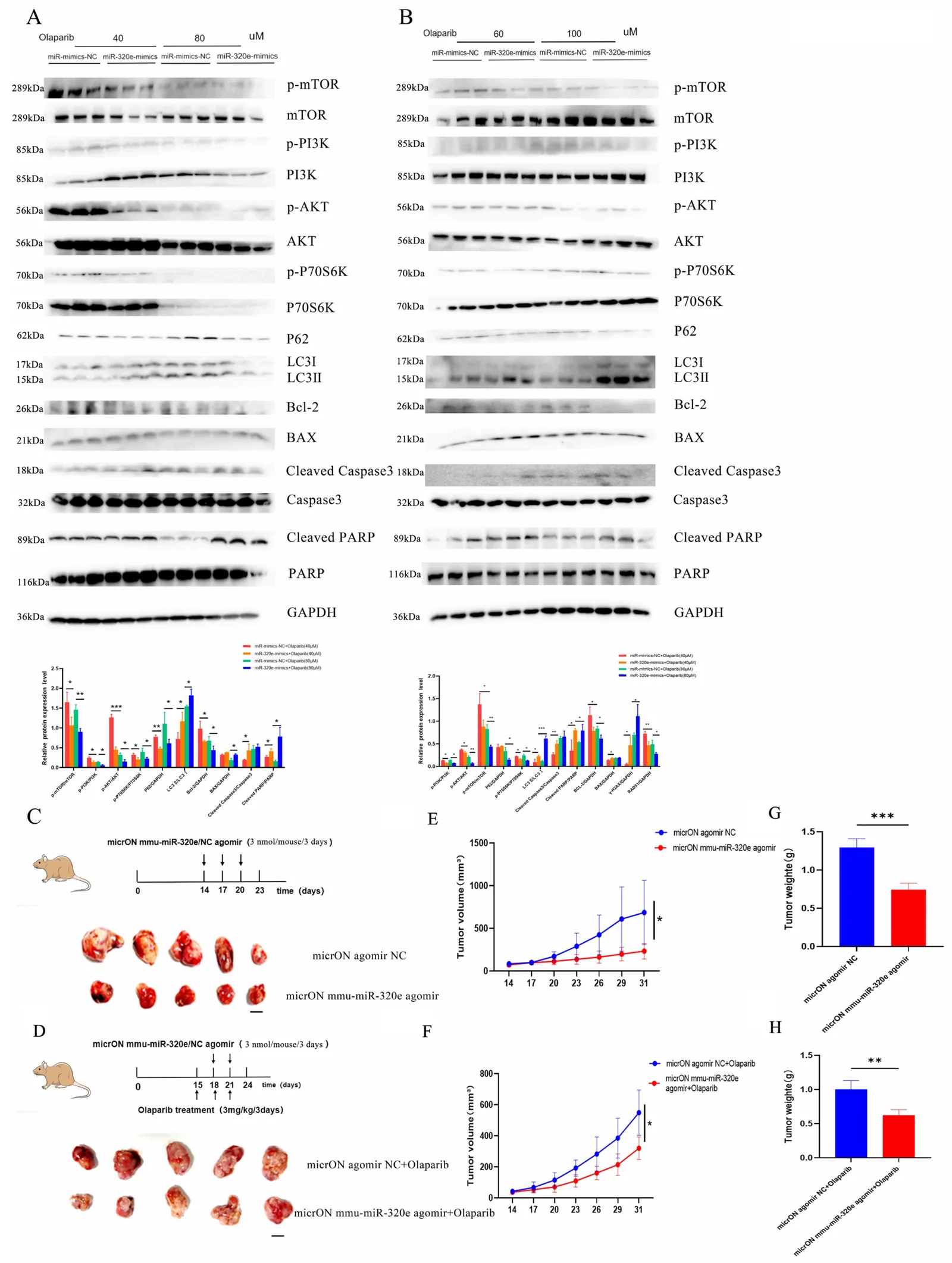
**MiR-320e overexpression enhances olaparib-mediated suppression of ovarian cancer progression.** (**A**) Compared with the control group, overexpression of miR-320e in A2780 cells significantly enhanced the inhibition of PI3K-AKT-mTOR pathway and the promotion of autophagy and apoptosis of olaparib at 40 μM and 80 μM. (**B**) Compared with the control group, overexpression of miR-320e in SKOV3 cells significantly enhanced the inhibition of PI3K-AKT-mTOR pathway and the promotion of autophagy and apoptosis of olaparib at 60 μM and 100 μM. Schematic illustration of nude mouse subcutaneous tumorigenesis model and isolated tumor images on day 23 and 29 (scale bar, 1 cm). (**C**) Indicates the group without olaparib combined treatment, while (**D**) indicates the olaparib combined treatment group. Tumor volume was measured every three days starting 10 days after subcutaneous inoculation to generate growth curves. (**E**) corresponds to the non-olaparib group and (**F**) to the combination treatment group, (**G**) and (**H**) separately show the weight of resected tumors from the two groups. Biological replicates (n = 3). *p < 0.05, **p < 0.01, ***p < 0.001.

WB experiments showed that transfection of miR-320e combined with olaparib had a significantly greater inhibitory effect on the PI3K-AKT-mTOR pathway in ovarian cancer cells compared to the combined effect of miR-mimics-NC and olaparib (all *p* < 0.05; Fig. 4A,B). The expression of P62, the LC3B II/LC3B I ratio, and corresponding apoptotic marker proteins was measured using WB assay (all *p* < 0.05; Fig. 4A,B), and the results showed that combining miR-320e mimics with olaparib could significantly enhance autophagy and apoptosis in ovarian cancer cells. Therefore, these results suggest that miR-320e significantly enhances ovarian cancer cell sensitivity to olaparib treatment by inhibiting the intracellular PI3K-AKT-mTOR signaling pathway and activating autophagy and apoptosis.

### 3.6 miR-320e Inhibits Tumor Growth and Increases Tumor Sensitivity to Olaparib In Vivo

To investigate the effects of miR-320e on ovarian cancer cell growth *in vivo* as well as chemotherapy resistance, we subcutaneously injected ovarian cancer cells into female nude mice. AgomiR is a chemically modified miRNA agonist known for its high stability and activity. AgomiRs are widely used to increase miRNA expression *in vivo* (Fig. 4C). Intratumoral injection of agomiR-320e or NC induced local expression of miR-320e in nude mice. The tumor growth curve showed that treatment with agomiR-320e significantly reduced tumor growth, tumor volume (*p* < 0.05; Fig. 4E), and mass (*p* < 0.001; Fig. 4G). To explore the effect of miR-320e on olaparib *in vivo*, we administered the drugs according to the schedule shown in Fig. 4D. The tumor growth curve showed that combined treatment with agomiR-320e and olaparib significantly inhibited subcutaneous tumor growth in mice (*p* < 0.05; Fig. 4F) and reduced mass (*p* < 0.01; Fig. 4H). These results indicate that miR-320e can enhance the inhibitory effect of olaparib on tumor growth *in vivo*.

### 3.7 FN1 Is a Downstream Target Gene of miR-320e

**Figure 5:**
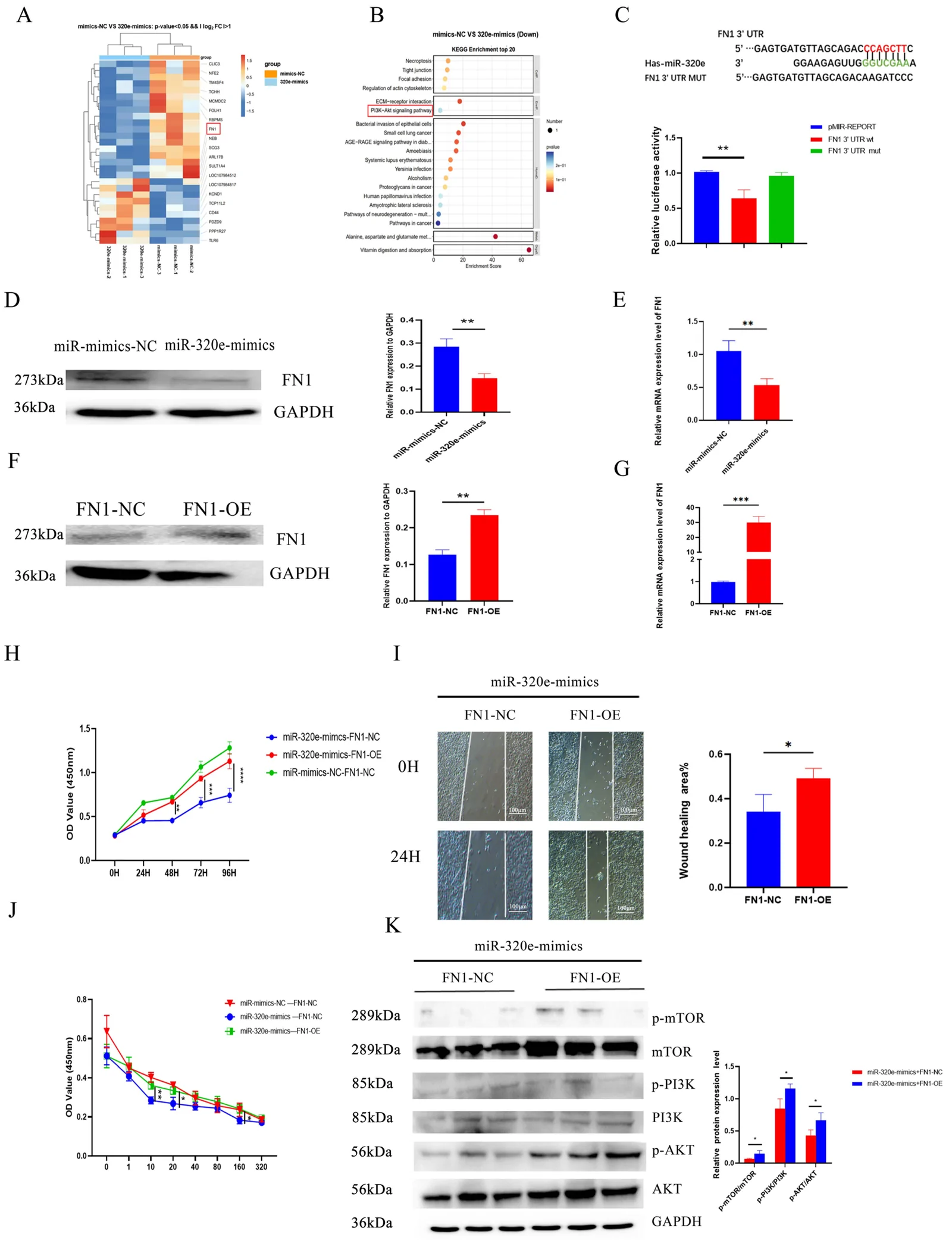
**FN1 is the downstream regulatory target of miR-320e.** (**A**) Heat map results showed that the expression of FN1 gene in ovarian cancer cells was significantly down-regulated after overexpression of miR-320e. The gene is highlighted with a red box. (**B**) The KEGG enrichment map showed that the PI3K-AKT signaling pathway was down-regulated in the treated group. The pathway is delineated by a red box. (**C**) Target Scan predicted that the FN1 3′-UTR has a miR-320e binding site, which is highly conserved in different species, and luciferase reporter assay showed that miR-320e directly targets the FN1 3′-UTR. 293T cells were co-transfected with FN1 3′-UTR-luciferase reporter, wild-type or mutant, and miR-320e mimic or miR-ctrl for analysis. 3′-UTR = 3′-untranslated region. wt = wild-type; mut = mutant. (**D**) The WB and (**E**) qPCR assay showed that the expression of FN1 in cells was significantly down-regulated after overexpression of miR-320e. (**F**) WB assay and (**G**) qPCR assay were used to verify the expression efficiency of FN1 after transfection with lentivirus. (**H**) CCK8 experiment demonstrated that overexpression of FN1 reduced the inhibitory effect of miR-320e on the proliferation of ovarian cancer cells. (**I**) Wound-healing experiments demonstrated that overexpression of FN1 reduced the inhibitory effect of miR-320e on the migration ability of ovarian cancer cells. (**J**) CCK8 experiment demonstrated that overexpression of FN1 reduced the increased sensitivity of ovarian cancer cells to olaparib due to overexpression of miR-320e. (**K**) WB experiment proved that overexpression of FN1 eliminated the inhibitory effect of miR-320e on PI3K-AKT-mTOR signaling pathway. Biological replicates (n = 3). *p < 0.05, **p < 0.01, ***p < 0.001, ****p < 0.0001.

To investigate how miR-320e specifically regulates the PI3K-AKT-mTOR pathway, we conducted RNA sequencing analysis on the transfected miR-320e group and the control group. Hierarchical clustering revealed that in A2780 cells, we detected 13,526 mRNAs. Compared to the control group, the transfection group showed higher levels of 10 transcripts and lower levels of 18 transcripts (log2-fold change > 1; adjusted *p* value (Q) < 0.05) (Fig. 5A). KEGG enrichment analysis demonstrated downregulation of the PI3K-AKT-mTOR signaling pathway in the treatment group, and the result is consistent with the WB experiment results in Section 3.2 (Fig. 5B). Based on previous studies, we speculated that FN1 played a role in affecting PI3K-AKT-mTOR among the significantly downregulated genes. Therefore, we predicted that FN1 is targeted by microRNA for regulatory purposes. Using TargetScan 8.0, we predicted that the 3′-UTR of FN1 mRNA contains a potential binding site for miR-320e. To determine if miR-320e regulates FN1 through binding to its 3′-UTR, we cloned the FN1 3′-UTR into the pmirGLO luciferase reporter vector and transfected it into 293T cells with either miR-320e mimic or miR-ctrl. Co-transfection of pMIR-REPORT-FN1-3′ UTR wt and miR-320e mimics resulted in decreased luciferase activity compared to miR-ctrl (*p* < 0.01; Fig. 5C). To further confirm specific regulation by miR-320e, we constructed a mutant version called pMIR-REPORT-FN1-3′ UTR mut, in which the seed sequence of the miRNA was deleted from the FN1-3′ UTR. Transfecting cells with miR-320e mimic or miR-ctrl and the mutant showed that deletion of the miR-320e binding site eliminated the effect on luciferase activity (Fig. 5C). In conclusion, these results confirmed that miR-320e specifically targets the FN1 3′-UTR, thereby inhibiting its expression. These findings indicate direct targeting of FN1 by miR-320e.

### 3.8 miR-320e Affects the PI3K-AKT-mTOR Signaling Pathway and Cell Proliferation and Migration by Regulating FN1

To investigate the role of FN1 in the regulation of the PI3K-AKT-mTOR pathway by miR-320e, miR-320e and nc-mimic were transfected, and the expression of FN1 was measured. Western blot (0.27 ± 0.08 vs. 0.14 ± 0.02, *p* < 0.01; Fig. 5D) and qPCR (1.05 ± 0.15 vs. 0.53 ± 0.10, *p* < 0.01; Fig. 5E) results showed decreased FN1 expression in the miR-320e mimic group compared to the control group. Next, the overexpression plasmid for the FN1 gene was constructed, and then a control plasmid and overexpression plasmid were transfected into A2780 cells (Fig. 5F,G). CCK8 and scratch assays were performed on co-transfected A2780 cells to further investigate the effect of FN1 overexpression on miR-320e. The results showed that A2780 cells overexpressing FN1 had recovered proliferation (0.74 ± 0.08 vs. 1.12 ± 0.08, *p* < 0.0001; Fig. 5H) and migration ability (0.34 ± 0.07 vs. 0.49 ± 0.04, *p* < 0.05; Fig. 5I) compared to the control group. Furthermore, CCK8 experimental results indicated that overexpression of FN1 reduces the sensitivity of A2780 cells to olaparib (Fig. 5J). WB assays showed that the PI3K-AKT-mTOR pathway was less inhibited in the FN1 gene overexpression plasmid group than in the control group (all *p* < 0.05; Fig. 5K). These results suggest that upregulation of FN1 can block the inhibitory effect of miR-320e on this pathway and can counteract the inhibitory effect of miR-320e on ovarian cancer cell proliferation and migration.

### 3.9 Fn1 Is Negatively Regulated by miR-320e In Vivo and Is Associated with the Malignancy of Ovarian Cancer

**Figure 6:**
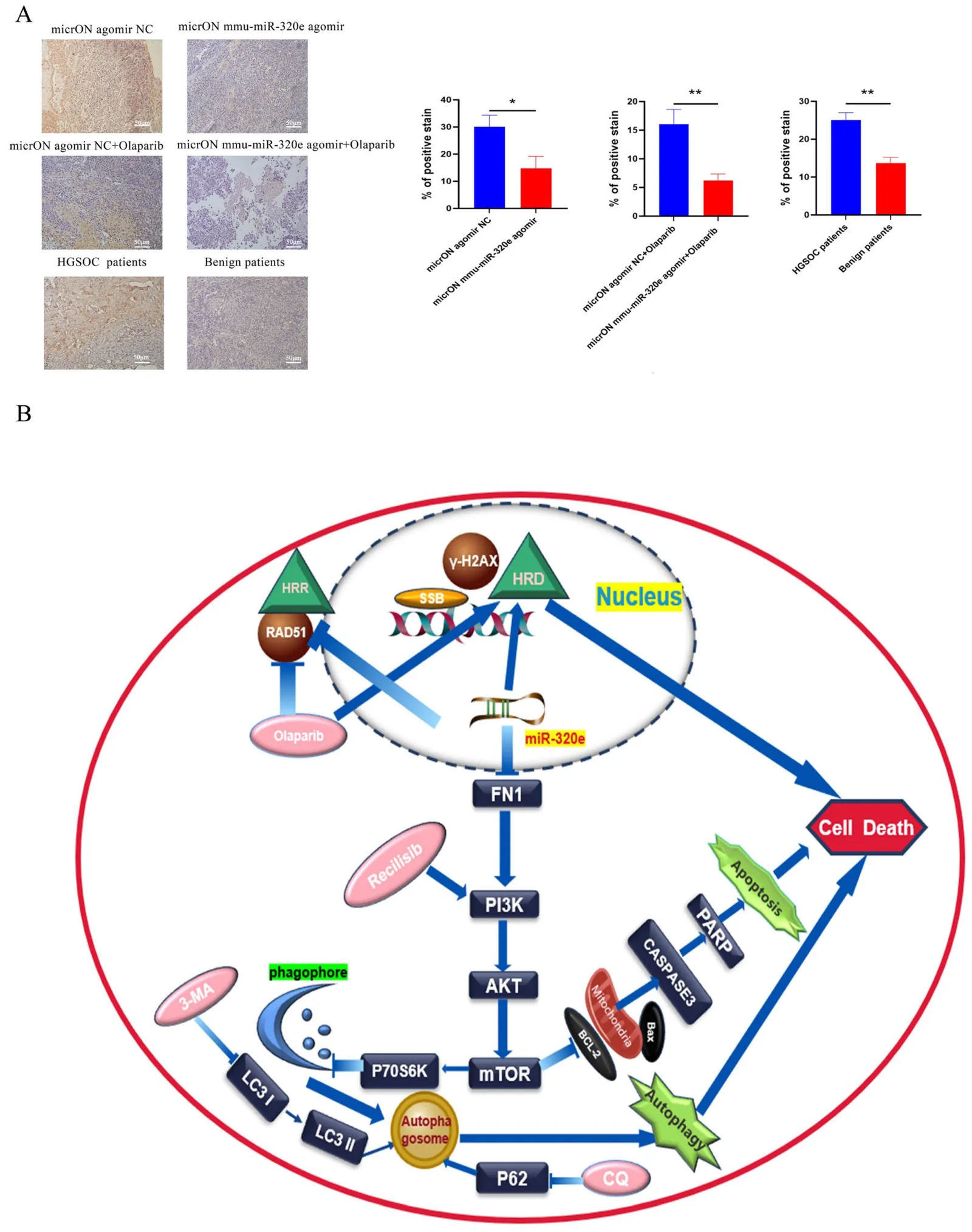
**Immunohistochemical results.** (**A**) The results of immunohistochemistry showed that the expression of FN1 was positively correlated with the malignant degree of ovarian cancer. (**B**) Pattern diagram of miR-320e regulatory mechanism. Biological replicates (n = 3). *p < 0.05, **p < 0.01.

Immunohistochemical results showed that the positive foci expression of FN1 in mouse tumor tissues was significantly reduced in the group treated with agomiR-320e alone or combined with olaparib compared to the control group (all *p* < 0.05; Fig. 6A). The results also showed that the number of FN1-positive lesions in the tissues of HGSOC patients was significantly higher than that in benign patients in immunohistochemical analysis. These results indicated that miR-320e can inhibit the expression of FN1 *in vivo*, and FN1 is correlated with the malignant degree of ovarian cancer. Finally, according to the experimental results, we drew a model diagram of miR-320e’s regulatory role in cells (Fig. 6B).

## 4 Discussion

In recent years, there has been extensive exploration and innovation in the treatment of ovarian cancer. Mutations in the BRCA1/2 gene lead to homologous recombination repair defects in ovarian cancer cells. As a result, the use of olaparib, which promotes “synthetic lethal” processes, is crucial in treating ovarian cancer. However, resistance to olaparib has emerged, making it imperative to discover new treatment options for ovarian cancer. Research has found that activation of the PI3K-AKT-mTOR signaling pathway is frequently observed during ovarian cancer development. Therefore, inhibiting this pathway’s activation to control disease progression has become a novel approach for treating ovarian cancer [21]. This pathway also plays a significant role in regulating autophagy—a cellular response that prevents cytotoxic protein accumulation and facilitates the recycling of materials for energy support [22]. Furthermore, studies have demonstrated that inhibiting this pathway can enhance tumor cell apoptosis and significantly impact the growth of ovarian cancer cells [23,24].

MicroRNAs, a class of regulatory RNA molecules, modulate gene expression and impact cellular functions. They can function as either carcinogenic factors or tumor suppressors, regulating downstream genes. miR-320e belongs to the miR-320 family, with miR-320a shown to negatively regulate prostate, breast, and colon cancer progression [25]. Studies have found that miR-320e is downregulated in submucosal colorectal carcinoma [26]. In this study, analyzing different ovarian cancer cell subtypes and patient tissues revealed low expression of miR-320e in HGSOC patient samples and high expression in benign patient samples, suggesting its potential as a therapeutic target for ovarian cancer. Transfecting miR-320e inhibited proliferation and migration while increasing apoptosis in A2780 and SKOV3 cells. Further analysis showed that overexpression of miR-320e suppressed intracellular PI3K-AKT-mTOR signaling pathways, affecting the proliferation, invasion, and migration of ovarian cancer cells. Inactivation of the PI3K-AKT-mTOR pathway activates downstream autophagy and promotes apoptosis [27], and this effect was blocked by corresponding inhibitors (PI3K pathway activator Recilisib; autophagy inhibitors 3-MA and CQ). These findings demonstrate that overexpression of miR-320e activates downstream autophagy to promote apoptosis while negatively regulating the proliferation and invasion of ovarian cancer cells. Increased γ-H2AX DNA damage marker expression alongside decreased RAD51 DNA repair marker expression further confirmed that miR-320e enhances DNA damage in ovarian cancer cells. Xenograft tumor growth experiments in mice confirmed significantly reduced tumor weight and volume upon transfection with miR-320e. These results indicate that miR-320e negatively regulates ovarian cancer cell proliferation and invasion both *in vivo* and *in vitro* by inhibiting the PI3K-AKT-mTOR pathway.

Previous studies have demonstrated that combining olaparib with PI3K-AKT-mTOR pathway inhibitors can enhance the inhibitory effect of olaparib alone [28]. We compared the results of ovarian cancer cells after treatment with olaparib and found that compared to the control group, the proliferation and migration ability of A2780 and SKOV3 cells in the miR-320e overexpression group was significantly reduced, and the degree of apoptosis was significantly increased, indicating that overexpression of miR-320e increased the sensitivity of ovarian cancer cells to olaparib. Through further analysis of the relevant pathways, we found that overexpression of miR-320e could enhance the inhibitory effect of olaparib on the PI3K-AKT-mTOR pathway in ovarian cancer cells, while promoting the activation of autophagy and the degree of apoptosis, and the regulatory effect of miR-320e on the above pathways was enhanced with increasing olaparib concentration. Accumulating evidence has demonstrated that miR-200c reverses Olaparib resistance in ovarian cancer cells via targeting NRP1 (Neuropilin 1) [29]. Building on these findings, our current work explored the function of miR-320e in modulating Olaparib resistance in A2780 cells, filling an important gap in the existing literature. Moreover, we examined the PI3K-AKT-mTOR pathway, which has been widely implicated as a critical mediator of drug resistance in ovarian cancer. In addition, the increased expression of γ-H2AX and decreased expression of RAD51 in the treatment group indicated that miR-320e could promote the “synthetic lethal” effect of olaparib on ovarian cancer cells. Xenograft tumor growth experiments in mice also confirmed that co-transfection of miR-320e with olaparib significantly reduced tumor weight and volume in mice compared to olaparib alone, and inhibition of the PI3K-AKT-mTOR pathway was also observed.

FN1 is a member of the glycoprotein family, widely expressed in various cells and playing a crucial role in cell adhesion and migration [30]. Studies have demonstrated its association with the occurrence and development of different cancers [31,32]. Serum levels of FN1 were found to be elevated in platinum-resistant patients [21]. In this study, RNA transcriptome sequencing revealed that FN1 was downregulated in the treatment group compared to the control group. This suggests that FN1 may be involved in regulating this pathway as a downstream target gene of miR-320e. Dual luciferase assay confirmed targeted binding sites between FN1 and miR-320e. Overexpression of FN1 weakened the inhibition of miR-320e on proliferation and migration and on the PI3K-AKT-mTOR signaling pathway. Immunohistochemistry and qPCR results indicated higher expression of FN1 in HGSOC tissues compared to benign cases and reduced amounts detected in subcutaneous tumors transfected with miR-320e. These findings confirmed that FN1 acts as a bridge between miR-320e and the PI3K-AKT-mTOR signaling pathway. The PI3K/Akt/mTOR pathway upregulates GBP2 (Guanylate binding protein 2)-mediated FN1 expression by activating STAT3, ultimately promoting the invasive phenotype of glioblastoma; FN1 is a key downstream effector molecule through which this pathway regulates tumor invasion [33]. Previous studies have linked AKT pathway activation induced by FN1 to peritoneal dissemination of ovarian cancer and platinum drug resistance [34].

Circulating miRNAs represent ideal non-invasive liquid biopsy biomarkers for ovarian cancer, as they are stable in serum and correlate with tumor progression and therapeutic response. The clinical application of miR-320e as a predictive biomarker for olaparib sensitivity relies on ultrasensitive detection technologies. Recent advances in enzyme-free electrochemical biosensors enable direct, femtomolar-level quantification of circulating miRNAs in patient sera without complex pre-processing (RNA extraction, reverse transcription, or PCR amplification) [35]. This technology allows rapid, cost-effective, and highly specific detection of serum miR-320e, which could be used to stratify ovarian cancer patients into olaparib-responsive and non-responsive subgroups, guide personalized PARP inhibitor therapy, and dynamically monitor treatment efficacy and disease recurrence. Thus, miR-320e holds dual translational value: as a therapeutic target to overcome olaparib resistance and as a circulating biomarker for precision medicine in ovarian cancer. Future studies will include long-term body weight monitoring, serum biochemical indexes, and histopathological examination of major organs to further evaluate systemic safety and off-target effects.

Although our study revealed the synergistic anti-tumor effect of miR-320e agonist combined with olaparib in ovarian cancer xenografts, the lack of systematic body weight and general health monitoring limited the full evaluation of potential off-target toxicity. Nonetheless, no obvious weight loss, lethargy, skin ulceration, or movement impairment was observed in mice during treatment, supporting acceptable tolerability of the combination at the tested doses. Future studies will incorporate body weight monitoring, serum biochemistry, and histopathological analysis of major organs to further assess systemic safety and off-target effects. In addition, since the PI3K/AKT/mTOR pathway closely interacts with MAPK, p53 and EMT signaling, further investigation is still required to clarify whether miR-320e exerts synergistic or independent regulation on these interconnected oncogenic networks. Expanding multi-pathway exploration will help to further deepen the systematic understanding of the anti-tumor mechanism of miR-320e.

In summary, our research provides new insights for treating ovarian cancer by transfecting miR-320e to inhibit the PI3K-AKT-mTOR signaling pathway, which suppresses the proliferation and invasion of ovarian cancer cells both *in vivo* and *in vitro*. Furthermore, the combination of miR-320e and olaparib enhances the “synthetic lethal” effect on ovarian cancer cells, leading to increased apoptosis and autophagy, and sensitizes ovarian cancer to olaparib therapy. Our study presents a novel approach for overcoming platinum resistance in ovarian cancer treatment.

## 5 Conclusion

This study proves that miR-320e can inhibit the PI3K-AKT-mTOR signaling pathway in A2780 and SKOV3 cells *in vivo* and *in vitro* through negative regulation of FN1, inhibiting the proliferation, migration, and invasion of ovarian cancer cells, and subsequently promoting cancer cell autophagy and apoptosis. Meanwhile, miR-320e can increase the sensitivity of ovarian cancer cells to olaparib therapy *in vivo* and *in vitro*.

## Data Availability

The datasets used and/or analysed during the current study are available from the corresponding author on reasonable request.

## Supplementary Materials

The supplementary material is available online at https://www.techscience.com/doi/10.32604/or.2026.081656/s1.
