# Supplementary material for: MicroRNA320e Augments the Synthetic Lethality of Olaparib by Regulating the PI3K-AKT-mTOR Pathway
Source: Oncol Res. 2026 Aug 13;34(9):27. doi: 10.32604/or.2026.081656 (PMC13494476; doi:10.32604/or.2026.081656)
Supplement: Supplementary file 1 [file OncolRes-34-81656-s001.zip › TSP_OR_81656-s001.docx]

**Table S1: Antibody Information for Western Blot**

| **Antibody** | **Dilution ratio** | **Company Name, City, Province/State, Country** | **Catalog number** |
| --- | --- | --- | --- |
| PI3K | 1:1000 | Cell Signaling Technology, Danvers, MA, USA | 4292S |
| p-PI3K | 1:1000 | Abcam, Cambridge, CB2 0AX, UK | ab302958 |
| AKT | 1:1000 | Cell Signaling Technology, Danvers, MA, USA | 9272 |
| p-AKT | 1:1000 | Cell Signaling Technology, Danvers, MA, USA | 4060S |
| mTOR | 1:1000 | Cell Signaling Technology, Danvers, MA, USA | 2972S |
| p-mTOR | 1:1000 | Cell Signaling Technology, Danvers, MA, USA | 5536S |
| GAPDH | 1:5000 | Cell Signaling Technology, Danvers, MA, USA | 2118S |
| P70S6K | 1:1000 | Cell Signaling Technology, Danvers, MA, USA | 34475S |
| p-P70S6K | 1:1000 | Cell Signaling Technology, Danvers, MA, USA | 9205S |
| LC3B | 1:1000 | Cell Signaling Technology, Danvers, MA, USA | 2775S |
| P62 | 1:1000 | Cell Signaling Technology, Danvers, MA, USA | 5114S |
| PARP | 1:1000 | Cell Signaling Technology, Danvers, MA, USA | 9542T |
| cleaved PARP | 1:1000 | Cell Signaling Technology, Danvers, MA, USA | 5625T |
| Caspase 3 | 1:1000 | Cell Signaling Technology, Danvers, MA, USA | 9662S |
| cleaved Caspase 3 | 1:1000 | Cell Signaling Technology, Danvers, MA, USA | 9661T |
| RAD51 | 1:1000 | Cell Signaling Technology, Danvers, MA, USA | 8875S |
| γ-H2AX | 1:1000 | Abcam, Cambridge, CB2 0AX, UK | ab81299 |
| Bcl-2 | 1:1000 | Abcam, Cambridge, CB2 0AX, UK | ab182858 |
| BAX | 1:1000 | Abcam, Cambridge, CB2 0AX, UK | ab182733 |
| FN1 | 1:1000 | Cell Signaling Technology, Danvers, MA, USA | 26836T |
| HRP-conjugated goat anti-mouse IgG (H+L) | 1:5000 | Beyotime Biotechnology, Shanghai, China | A0216 |
| HRP-conjugated goat anti-rabbit IgG (H+L) | 1:5000 | Beyotime Biotechnology, Shanghai, China | A0208 |
